# MEDICAID POLICY AND ASSISTED LIVING ACCESS

**DOI:** 10.1093/geroni/igac059.1652

**Published:** 2022-12-20

**Authors:** Lindsey Smith

**Affiliations:** Brown University, Providence, Rhode Island, United States

## Abstract

Funding for home and community-based services (HCBS), including assisted living (AL), using Medicaid is limited to either Medicaid program waivers or state plan amendments. The Affordable Care Act (ACA) created a new option for the state plan, the Community First Choice 1915(K), that mandates all care is self-directed and inclusive of families in decision making while disallowing waiting lists. This presentation will provide an overview of the various combinations of Medicaid waivers and amendments used by states to cover services in AL. We will then share the results of a study that used coincidence analysis, a configurational comparative method, to compare states’ approaches to HCBS Medicaid coverage and a measure of AL geographic access. We found that both Medicaid waivers and 1915(K) amendments are associated with increased geographic access to AL.

